# Measuring the impact of methodological research: a framework and methods to identify evidence of impact

**DOI:** 10.1186/1745-6215-15-464

**Published:** 2014-11-27

**Authors:** Valerie C Brueton, Claire L Vale, Babak Choodari-Oskooei, Rachel Jinks, Jayne F Tierney

**Affiliations:** Medical Research Council Clinical Trials Unit at University College London, Aviation House, 125 Kingsway, London, WC2B 6NH UK

**Keywords:** Framework, impact measurement, methodological research, methodology

## Abstract

**Background:**

Providing evidence of impact highlights the benefits of medical research to society. Such evidence is increasingly requested by research funders and commonly relies on citation analysis. However, other indicators may be more informative. Although frameworks to demonstrate the impact of clinical research have been reported, no complementary framework exists for methodological research. Therefore, we assessed the impact of methodological research projects conducted or completed between 2009 and 2012 at the UK Medical Research Council Clinical Trials Unit Hub for Trials Methodology Research Hub, with a view to developing an appropriate framework.

**Methods:**

Various approaches to the collection of data on research impact were employed. Citation rates were obtained using Web of Science (http://www.webofknowledge.com/) and analyzed descriptively. Semistructured interviews were conducted to obtain information on the rates of different types of research output that indicated impact for each project. Results were then pooled across all projects. Finally, email queries pertaining to methodology projects were collected retrospectively and their content analyzed.

**Results:**

Simple citation analysis established the citation rates per year since publication for 74 methodological publications; however, further detailed analysis revealed more about the potential influence of these citations. Interviews that spanned 20 individual research projects demonstrated a variety of types of impact not otherwise collated, for example, applications and further developments of the research; release of software and provision of guidance materials to facilitate uptake; formation of new collaborations and broad dissemination. Finally, 194 email queries relating to 6 methodological projects were received from 170 individuals across 23 countries. They provided further evidence that the methodologies were impacting on research and research practice, both nationally and internationally. We have used the information gathered in this study to adapt an existing framework for impact of clinical research for use in methodological research.

**Conclusions:**

Gathering evidence on research impact of methodological research from a variety of sources has enabled us to obtain multiple indicators and thus to demonstrate broad impacts of methodological research. The adapted framework developed can be applied to future methodological research and thus provides a tool for methodologists to better assess and report research impacts.

**Electronic supplementary material:**

The online version of this article (doi:10.1186/1745-6215-15-464) contains supplementary material, which is available to authorized users.

## Background

For the benefits of research to be demonstrated to society, researchers are increasingly being asked to demonstrate not just the outputs of their research, but also measures of its impact. For example, many funding bodies require researchers to demonstrate the impacts of their completed research, whilst others, including the UK Medical Research Council (MRC), expect all new funding applications to outline their plans to measure impact. Approaches to demonstrating and measuring the impact of clinical research have been reported [1–3] and a framework has been proposed [4]. This framework, the Becker model, categorizes different types of research output, such as development of collaborations or different ways of disseminating research to demonstrate such impacts as advancement of knowledge and implementation.

In 2008, the UK MRC Clinical Trials Unit (CTU) became one of eight regional hubs for trials methodology research. Methodological research conducted by the MRC CTU Hub is primarily designed to develop research methods, to improve the quality and consistency of research practice in three areas: applied statistical methodology, trial-conduct methodology and meta-analysis methodology. Guidance on the application of the methods developed is also provided, to improve the quality and reliability of research both within the MRC CTU and elsewhere. There is also a need to demonstrate the impact of methodological research, but, to our knowledge, no tool exists for the measurement of such impact. Therefore, we aimed to identify ways to assess indicators of impacts beyond simply identifying publication and citation rates of our research.

## Methods

To quantify a variety of impact indicators, we used three separate approaches. The first of these was focused on standard indicators relating to publications and citations of published research. We also used interviews to explore other indicators, such as collaborations, wider dissemination and knowledge transfer. Finally, we sought evidence of implementation of the research, both through the interviews and also through the analysis of email queries. Application of the NHS REC Health Research Authority decision-making tool [5] indicated that because our research was not a clinical trial of a medicinal product or device; no clinical data were collected and the interviewees were neither trial participants nor NHS patients, ethics approval was not necessary (Additional file 1) and on this basis, was not sought. The study did not receive specific funding, but was conducted in the full knowledge of the MRC CTU senior management group. Furthermore, the researchers who agreed to participate in the interviews to evaluate impact of the Unit’s methodological research are fully aware and supportive of the results being written up for publication.

### Citation analysis

We identified all CTU methodology publications in peer-reviewed journals dating from 01 January 2009 to 31 December 2012. For each eligible publication, we extracted the full publication and journal title, journal impact factor, publication date, and the theme of the methodological research (for example, applied statistical methodology). This information was logged, along with citation counts obtained from the Web of Science [6], and is up to date as of 11 December 2013. We also used the Web of Science to explore the citations of the eligible publications, for example assessing whether the citations were found in original research (for example, a clinical trial publication) or review articles (for example, a review of different methodologies). We also noted the clinical or academic discipline of the citing articles. To allow for the range of publication dates, average annual citation rates for each publication were calculated from the total number of citations and the date of first publication (either online or in print). Publications that were too recent to have been included, and those published in journals that are not catalogued in Web of Science, were necessarily excluded from these calculations. Journals were grouped into broad categories, such as statistics, clinical trials and general medical for ease of analysis. The data were exported into Stata [7] for descriptive statistical analysis.

### Interviews

We identified a sampling frame of ongoing or completed methodology projects for the period January 2009 to December 2012 and a corresponding sample of the MRC CTU Hub methodologists who had led these projects. Each methodologist was invited for interview via email. Information about the study aims, focus and interview length was provided in the invitation email. Each interviewee gave written permission via email to be interviewed; this was taken as consent to study participation. Each interview was held at a time and place convenient to the participant. The interviews were conducted by one author with interview experience (VCB) at a place and time convenient to the methodologist.

To obtain quantitative information on the frequency and variety of indicators of the impacts of each methodological research project, we developed a semistructured interview schedule. The schedule included questions on dissemination, production of software or guidance documents, teaching and workshops and changes in practice relating to the research (Additional file 2). Interviewees were asked to identify the number of impacts, if any, under each category. As peer-reviewed articles were examined separately, we purposefully did not ask about publications during the interview. Pilot interviews using the draft interview schedule were conducted with two methodologists. The schedule was subsequently revised and finalized following one further pilot interview. Data from pilot interviews were included in the final dataset for analysis. Contemporaneous written notes of each interview were taken by the interviewer (VCB) and a second author (CLV). The interviews were not recorded. The notes were checked and any queries were resolved between two authors (VCB, CLV). Unresolved data queries were sent back to the interviewee to be resolved and final summaries of each interview were returned to each interviewee for validation. Finally, data for each unique methodology project were extracted from verified interview notes using a standard data extraction form and entered into a Microsoft Excel spreadsheet for data management. For some projects, this meant combining quantitative data from multiple interviewees. Final data for each project were analyzed using Stata [7]. For the analysis, some additional grouping was made for questions about geographic location (for example, location of collaborators or conferences, or where new methods developed have been adopted), the answers were grouped as: internal, national (within the UK) or international (outside of the UK). Where multiple locations were given, the furthest afield was used to define the category. The data for meetings, workshops and lectures were combined into one variable for reporting, as it was felt that there was considerable overlap in how they were described by the interviewees.

### Email analysis

Methodologists who indicated that they received email queries about their research were asked to supply these emails to a central mail box. The content of each email was evaluated by two authors (CLV, BC-O). Data on the subject of the email, the date received, and the location and institution of the individual initiating the query were recorded for descriptive analysis.

## Results

### Publications and citations

**Figure 1:**
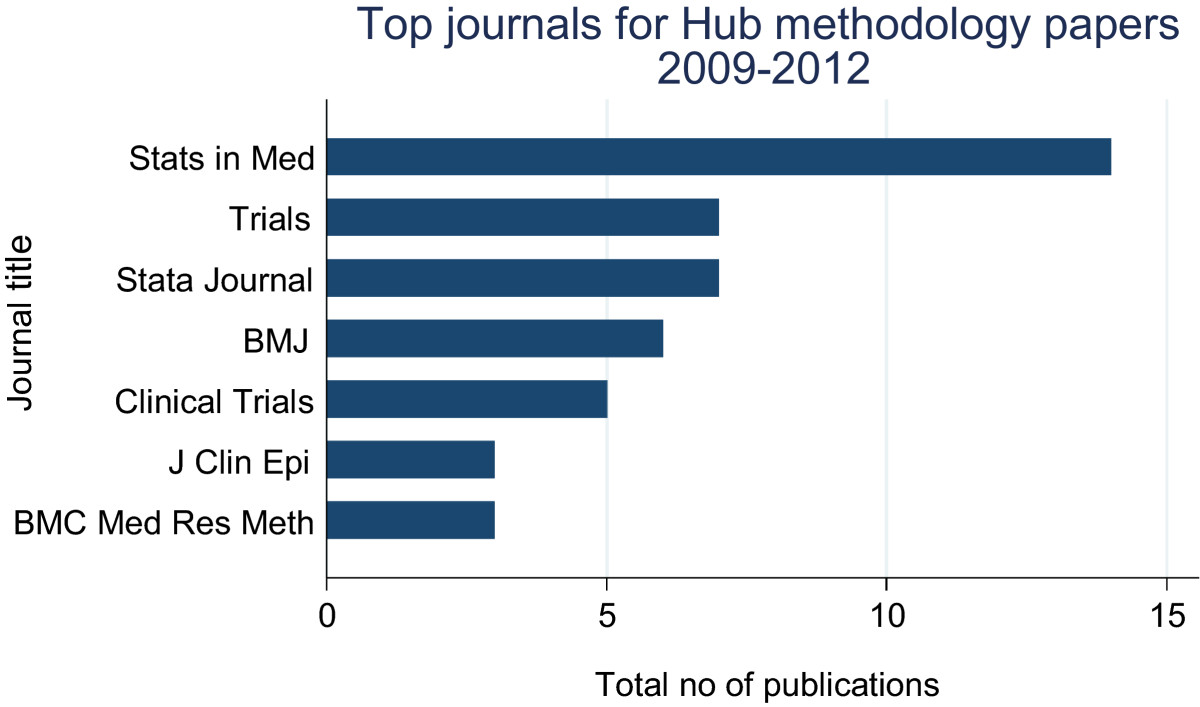
**Journals in which MRC CTU methodology papers were most frequently published, 2009 to 2012 (top 63% of all articles).**

**Figure 2:**
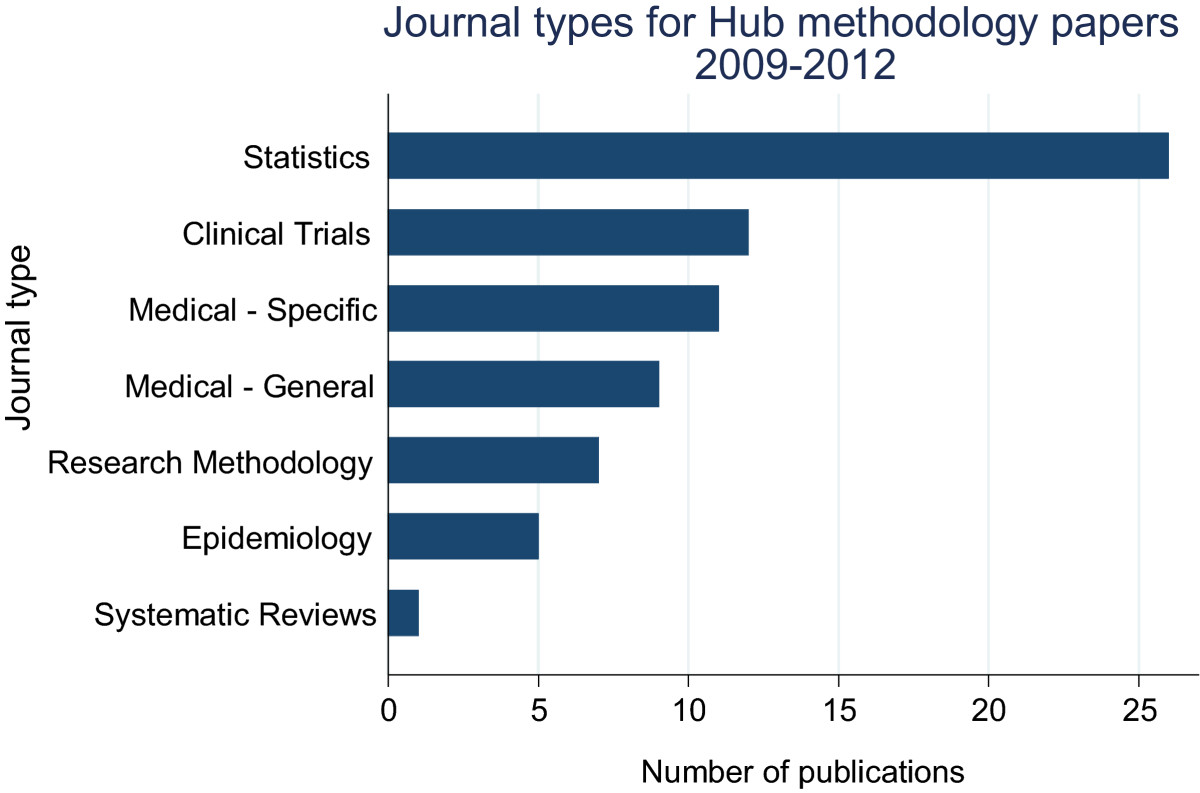
**Journal types publishing MRC CTU Hub methodology papers (2009 to 2012).**

**Figure 3:**
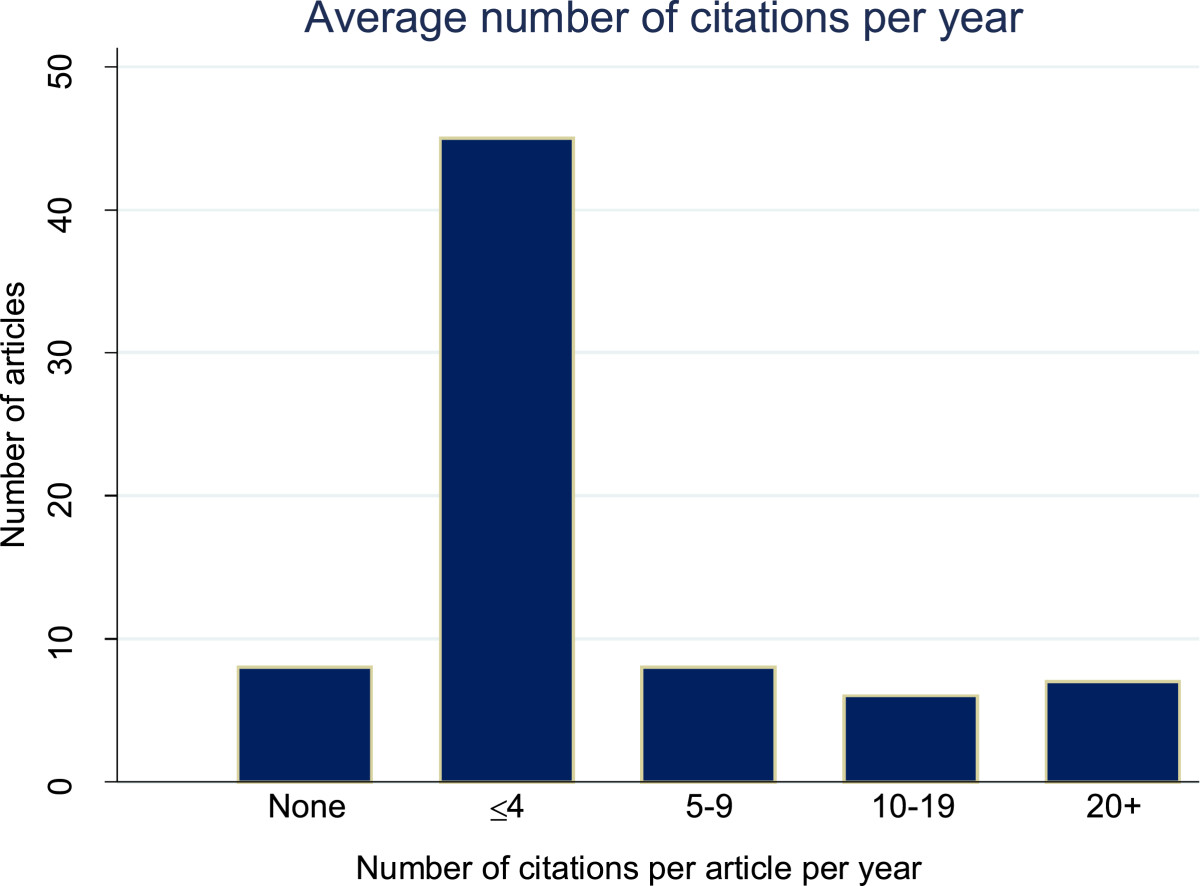
**Average number of citations per publication per year (2009 to 2013).**

We identified 74 eligible peer-reviewed publications, most of which were published in either statistics journals (*n* = 26), clinical trials journals (*n* = 12), disease-specific or general medical journals (*n* = 20, Figures 1 and 2). Eight articles (11%) had not yet been cited, however most of these had only been published in 2012. More than half of all publications (*n* = 43, 58%) had been cited fewer than four times per year, with the remaining publications (*n* = 23, 31%) having been cited more frequently (Figure 3).

More detailed mapping of the four most highly cited publications showed that these four articles [8–11] were subsequently cited in original research articles (for example, clinical trials, cohort studies), and in review articles, editorials and letters spanning a number of fields, including general internal medicine, cardiovascular disease, cancer, and orthopaedics.

### Interviews

Fourteen interviews were conducted with methodologists at the MRC CTU hHub between June and October 2012. The interviewees spanned the three research themes and were linked to 20 individual projects. Most were in the area of statistical methodology (*n* = 13), with the remaining projects representing trial-conduct methodology (*n* = 6) and meta-analysis methodology (*n* = 1, Table 1). For three projects (for example, the multi-arm multi-stage trial design), information came from several interviewees (Table 1).

**Table 1:** **Methodology projects**

Project title	Methodology area	Interviewee number*
Flexible parametric models	Statistical	13
Missing data for prognostics	Statistical	13
Multi-variable modelling, prognostic modelling	Statistical	13
Modelling the association between patient characteristics and change over time in a disease measure	Statistical	3
Combining multiple imputation and inverse probability weighting	Statistical	3
New measure of the predictive ability for a survival model	Statistical	7
Bias two-arm multi-stage trials	Statistical	7
Analysis of resources for trials	Statistical	8
Restricted mean survival time	Statistical	4
Multi-arm multi-stage trial design	Statistical	5, 10
Biomarkers	Statistical	4
Comparing dynamic treatment regimens or monitoring strategies	Statistical	11, 12
Estimating the effect of time-varying treatment or exposure on outcome	Statistical	11, 12
Developing guidance for researchers on patient and public involvement in clinical research	Trial conduct	2
Consumer involvement in MRC CTU studies	Trial conduct	2
DAMOCLES (DAta MOnitoring Committees: Lessons, Ethics, Statistics)	Trial conduct	5
Retention strategies for randomized trials	Trial conduct	14
Central monitoring techniques to replace on-site monitoring	Trial conduct	6
Risk-based monitoring of trials	Trial conduct	9
Analysis of subgroup interactions in individual patient data meta-analysis	Meta-analysis	1
**Total**	20	14

*Interviewees 2, 3, 4, 5, 7, 11, 12 and 13 discussed more than one project. CTU, clinical trials unit; MRC, UK Medical Research Council.

Table 2 summarizes research outputs for the 20 projects identified. These projects led to several conference presentations, lectures and collaborations.

**Table 2:** **Research outputs identified through interviews**

Research output	Total	Range per project	Median per project
Meetings	1	—	—
Workshops	10	1 to 3	1
Lectures	32	1 to 10	2
Conference presentations	42	1 to 18	2

### Dissemination

#### Books and grey literature publications

Two books relating to statistical methodology were published within the timeframe. In addition, three pieces of online guidance were produced, two on involvement of patients and the public in clinical trials and systematic reviews [12, 13] and the third on the assessment of risk in the management and monitoring of clinical trials [14].

#### Conference presentations

In total, there were 42 presentations at both national and international conferences relating to 14 of the 20 projects (70%, Table 2). Five projects were presented at national conferences only, five projects were presented at international conferences and four projects were presented at both national and international conferences. Conferences were usually methodology-themed (for example, the International Society for Clinical Biostatistics and the UK Clinical Trials Methodology conference) or disease-specific (for example, the British Gynaecological Cancer Society). Six projects had not yet been presented at any conferences. It was unclear whether they were likely to be presented in the future.

In addition to presentations at conferences, 17 of 20 (85%) projects had been presented or discussed in lectures, meetings or workshops between 2009 and 2012, mostly (*n* = 16, 94%) outside of the MRC CTU. Three statistical methodology projects had been included in more than 19 meetings, lectures or workshops within the timeframe.

#### Software and training materials produced

Of the 20 included projects, 8 (40%) had developed statistical software (Stata [7]); 6 (30%) had produced training materials and 3 (15%) had produced both software and guidance to enable the methodology to be better applied. For eight of the projects that had developed software that had been made freely available, interviewees noted that these were being commonly used, both within the MRC CTU Hub and externally.

### Impacts on methodological research or practice

Of the 20 projects 12 (60%) provided evidence that they had influenced changes in research practice. Five projects reported changing research practice within the MRC CTU Huband a further seven projects reported changing practice outside the MRC CTU Hub. All of the projects that had produced statistical software or training materials (*n* = 8) were reported to have changed practice at the MRC CTU, and these methods were also currently being applied in ongoing projects being run through the MRC CTU. Five projects that had not produced software or training materials also reported changes to the way data were analyzed. For the remaining three projects, it was thought to be too early to show any evidence of a change in practice or it was unclear at the time of interview whether practice had changed.

### Methodology projects leading to new research or collaborations

Methodologists reported that over half of the methodology projects (*n* = 12) led to a new research project. The new follow-on projects reported were mainly in the area of statistical methodology (*n* = 8), with fewer in trial-conduct methodology (*n* = 3) or meta-analysis methodology (*n* = 1). Furthermore, five statistical methodology projects had led to PhD studentships to further develop the methodology.

Methodologists also reported 29 external collaborations relating to 12 of the 20 projects. The majority of these 12 projects had three collaborations or fewer. Collaborations were largely with other researchers based within the UK; however, four projects had led to international collaborations with research institutions and universities in France, Germany, Netherlands and the USA.

### Email analysis

Most of the methodologists interviewed had not received email queries about their work; however four methodologists were able to supply them for this analysis. As the emails were collected retrospectively, and had not necessarily been saved systematically by the four individuals, they did not cover the whole time period from January 2009 to December 2012. However, the available email data comprised 194 queries received from 170 individuals across 23 countries in Europe, North America, Asia and beyond. The queries related to six research projects and commonly sought clarification about application of the specific method, or use of accompanying computer software. Some emails requested further information or advice relating to further development or novel applications of the method.

### Developing a framework for reporting impact: case study

**Figure 4:**
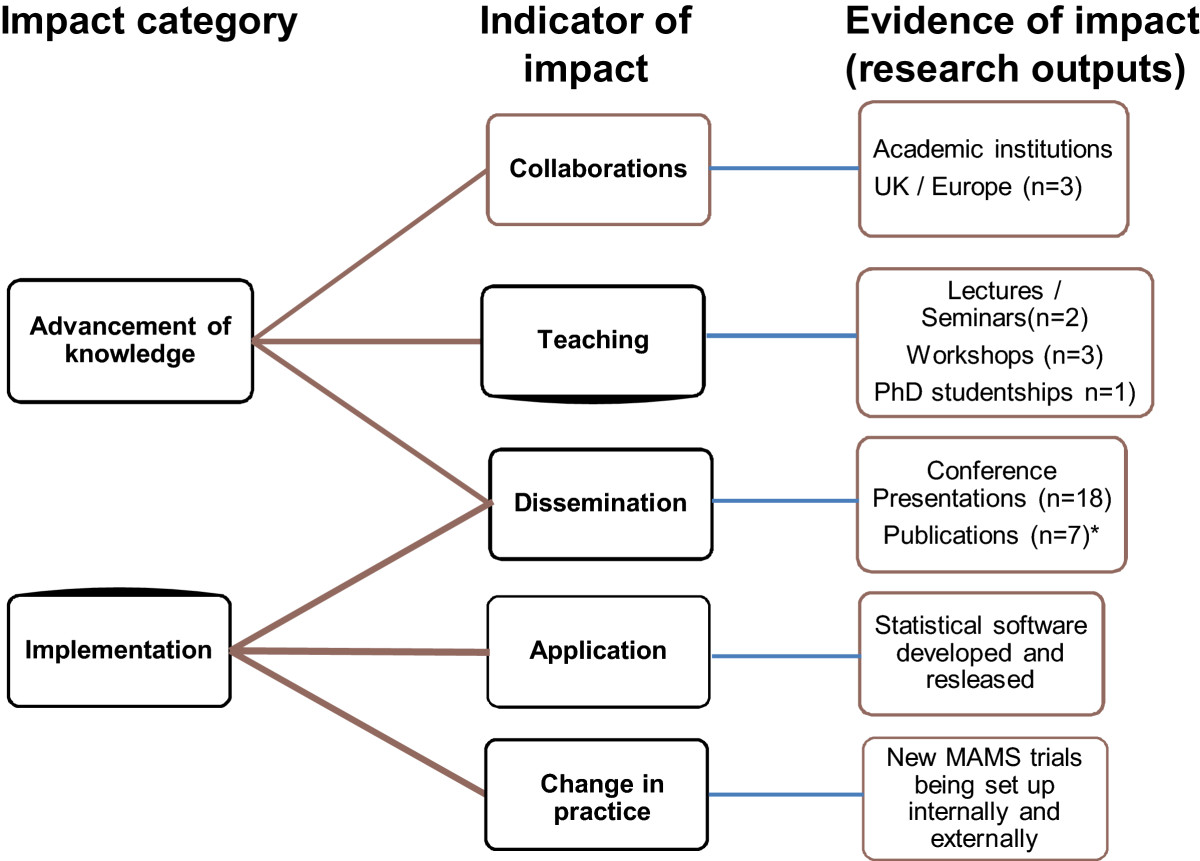
**Impacts for the MRC CTU Hub multi-arm multi-stage trial design 2009 to 2012.** *Publications from 2008 to 2013 only.

Using the three methods, we identified a range of key indicators that illustrate the impact of our methodological research and have used them to develop a framework, based on the Becker model [4]. The collection of evidence of collaboration, teaching (lectures, workshops and PhD studentships), dissemination (conference presentations and publications), applications (statistical software and guidance documents) and changes in research practice (that is, examples of trials from within the MRC CTU and from other organizations that have adopted this design) has enabled us to demonstrate a range of impacts, including research outputs and activities; advancement of knowledge, and implementation. Figure 4 shows this framework and summarizes the impacts attributable to the multi-arm multi-stage trial design project [15–17]. This framework will now be used to collate and better report impact for future and ongoing methodology studies.

## Discussion

The use of three approaches illustrated a variety of impact indicators in addition to numbers of publications and subsequent citations. The rich quality of information gathered from interviews with methodologists enabled us to better identify indicators of impact that had not otherwise been collated, such as advancement of knowledge (illustrated through collaboration, workshops and seminars) and implementation (demonstrated by the application of new methods within new trials). The implementation of methodological research was aided by the provision of statistical software, training materials and books. Content analysis of email queries received by some methodologists also gave a broad demonstration of the methodological research being used in further research and practice.

Collating information about methodological research outputs and linking these outputs to the different types of impact they indicate has allowed us to better understand the impacts of our methodological research to date. Obviously, these data are not exhaustive, being limited only to projects being led within the MRC CTU. Furthermore, because the data were retrospective, they might not always be complete or consistent. In addition, data from the three approaches does not always link to the same group of projects; for example, publications included in the sample relate to many more projects than the 20 projects for which interviews were conducted. Similarly, the data from emails relate to only six projects in total. However, having identified the value in collecting these data, we hope to move towards developing a systematic prospective collection of a set of core routine data for all projects.

We are not aware of any other prior work that aimed to assess the impact of methodology research, neither could we identify any model or framework for assessing such impacts. However, similar methods to the multi-method approach that we used have been used for clinical research [4], and resulted in a framework, the Becker model. This was designed to measure impact, demonstrated through research outputs and other indicators of clinical research, in key themes, including research outputs, advancement of knowledge, implementation of findings, community benefit, legislation and policy, and economic benefit. Whilst perhaps not all of these impacts apply to methodological research, there are some areas of overlap. We found evidence from our three approaches to support impact relating to three areas included in the Becker model: research output, advancement of knowledge and implementation of findings. We therefore suggest this as a basic framework for demonstrating impact of methodological research, as shown in the example of the multi-arm multi-stage trial design.

An increasing number of public sector and academic funders now require evidence of research impact. The methods we used provided a useful way of identifying broader application of the research methodologies beyond citation analysis. In addition, measuring the impact of our research has enabled us to review the projects with the highest impacts and to learn from these. For example, we found that all of the projects that released freely available accompanying software could demonstrate implementation through the use of the software, which had enabled others to readily apply the methodology. We are currently investigating ways to optimise impact across all areas of methodological research.

## Conclusions

Gathering evidence through analysis of publications and their citations, semistructured interviews and analysis of research queries enabled us to obtain multiple indicators and thus to demonstrate broad impacts of methodological research. Collating evidence of impact has enabled us to adapt a framework that may be broadly applied to future methodological research; provide reports to funders; demonstrate impact of our research more broadly and, finally, be applied by other researchers.

## Electronic supplementary material

Additional file 1: Ethical approval decision tool results.(PDF 37 KB)

Additional file 2: Topic guide used to explore the impact of MRC CTU Hub methodology research.(PDF 25 KB)

Below are the links to the authors’ original submitted files for images.

Authors’ original file for figure 1

Authors’ original file for figure 2

Authors’ original file for figure 3

Authors’ original file for figure 4

## Abbreviations

CTU: clinical trials unit
MRC: Medical Research Council.
