# Supplementary material for: Measuring the impact of methodological research: a framework and methods to identify evidence of impact
Source: Trials. 2014 Nov 27;15:464. doi: 10.1186/1745-6215-15-464 (PMC4258950; doi:10.1186/1745-6215-15-464)
Supplement: Supplementary file 2 — Additional file 2: Topic guide used to explore the impact of MRC CTU Hub methodology research.(PDF 25 KB) [file 13063_2014_2328_MOESM2_ESM.pdf]

1. Looking at hub reports since 2008 I have a list of the projects you have been involved with here:

(Give interviewee a copy of the list)

	Methodology projects	Tick biggest impact
1		
2		
3		

1a Have we missed any projects from this list?

YES ☐ **Which ones are missing?**
(Add missing ones to the list)
(Then go to next question no 1b)

NO ☐ *(Go to next question no 1b)*

1b Which of these projects has had the biggest impact so far?
(Tick the one with the biggest impact).
(Then go to next question no 1c)

******1c Thinking about the xxxxx project first, Can you give us more information about that project, in particular a <u>brief</u> rationale for why it was developed?**
(Then go to next question no 2)

2. Has it become standard practice to use this method in CTU?

NO ☐ *(Skip to question no 2a)*

YES ☐ *(Go to next question no 2b)*

2a Has it been used in any CTU studies

NO ☐ *(Skip to question no 3)*

YES ☐ *(Go to next question no 2b)*

2b Can you tell me about the studies it is/was applied to in CTU? *(Go to next question 2c)*

	Study
1	
2	
3	

2c <u>What was the benefit of</u> using this method in each study?
(Go to next question no 3)

Study	Benefit
1	
2	
3	

Use of method outside CTU

3 Are you aware of the method being used <u>outside CTU</u>?

NO ☐ *(Skip to question no 4)*

YES ☐ *(Go to next question no 3a)*

3a Do you know where it is being used? *(Which groups and studies)*
(Then go to next question no 4)

	Group	Study
1		
2		
3		

4 Has there been any software developed to accompany the method?

NO ☐ *(Skip to question 5)*

YES ☐ *(Go to next question no 4a)*

4a Can you tell me what that software does?
(Go to next question no 4a1)

4a1) Is it a Stata program?

NO ☐ *(Go to question no 4b)* **YES** ☐ *(Go to next question no 4a2)*

4a2) If YES, ask has it become a standard command in Stata?

NO ☐ *(Go to next question no 4b)* **YES** ☐ *(Go to next question no 4b)*

4b Is the software used in CTU?

NO ☐ *(Go to next question no 4c)* **YES** ☐ *(Go to question no 4c)*

4c Are there any plans to roll it out?

NO ☐ *(Skip to question no 4d)* **YES** ☐ *(Go to next question no 4c1)*

4c1 Where? *e.g.*

Publish in STATA journal **NO** ☐ **YES** ☐
(Then ask next question)

Can we include it as a resource in the methodology website? **NO** ☐ **YES** ☐
(Go to next question no 4d)

4d Does anyone else use it?

NO ☐ *(Go to next question no 5)*
YES ☐

Who?
(Then Go to next question no 5)

5 Has the method been further developed?

NO ☐ *(Go to next question no 5a)* **YES** ☐ *(Skip to question no 5b)*

5a Are there any plans to develop it?

NO ☐ *(Skip to question no 6)* **YES** ☐ *(Go to next question no 5b)*

5b What developments have been made or are planned?
(Go to next question no 5C)

5c) Where?
CTU ☐ *(Go to next question no 5d)* **External** ☐ *(Go to next question no 5d)*

5d) By whom? *(Go to next question no 6)*

6 Has use of the method to any new collaboration/s outside CTU? e.g. in particular, any new funding/grant applications
(Go to next question no 6a)

NO ☐ *(Skip to question no 7)* **YES** ☐ *(Go to next question no 6a)*

6a Can you tell me more about those?
(Go through each one) (Then go to next question no 7)

Developments	Collaboration	Funding/grant applications
1		
2		

We already know about your presentations and publications but

7 Are there any other ways that you have disseminated your method? E.g. conferences, text books, websites, workshops etc.
(Then go to next question no 8)

8 Are you aware that it has become included in any teaching materials e.g. training you have developed or part of MSc courses modules etc.?

NO ☐ *(Skip to question no 9)* **YES** ☐ *(Then go to next question no 8a)*

8a By whom? *(Then go to next question no 8b)*

8b Which course?
(If there are no more methods listed in question 1 then go to next question no 9)

If there were more methods listed in Question 1 go through the following.

We have concentrated on X method
Now can we focus on Y method

Repeat all the above questions from question 1c ** to 8b**
(Then go to next question no 9)

9 Do you or anybody else that worked on the project get emails enquiries about the method you developed?

If **NO**

Thank the interviewee for their time
Tell them if they think of anything else to contact you
Ask if we can contact them with any queries

If **YES**
Ask if it is ok to set them up with this and explain how it works

Thank them very much for their time
Ask if we can contact them again for further info?
