# Supplementary figures and images for: Measuring the impact of methodological research: a framework and methods to identify evidence of impact
Source: Trials. 2014 Nov 27;15:464. doi: 10.1186/1745-6215-15-464 (PMC4258950; doi:10.1186/1745-6215-15-464)

# Top journals for Hub methodology papers 2009-2012

Journal title

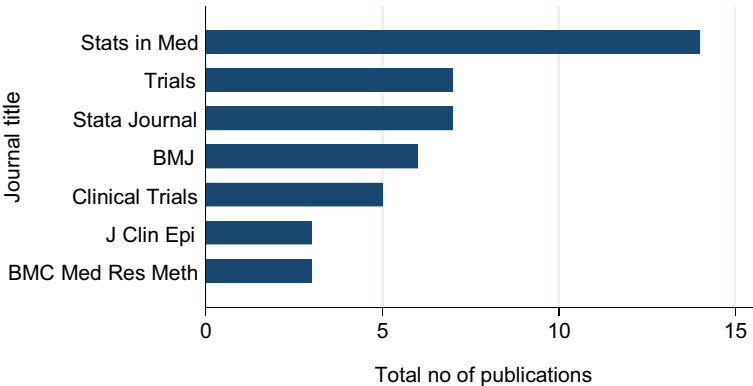

Supplement: Supplementary file 3 — Authors’ original file for figure 1 [file 13063_2014_2328_MOESM3_ESM.pdf]

## Journal types for Hub methodology papers 2009-2012

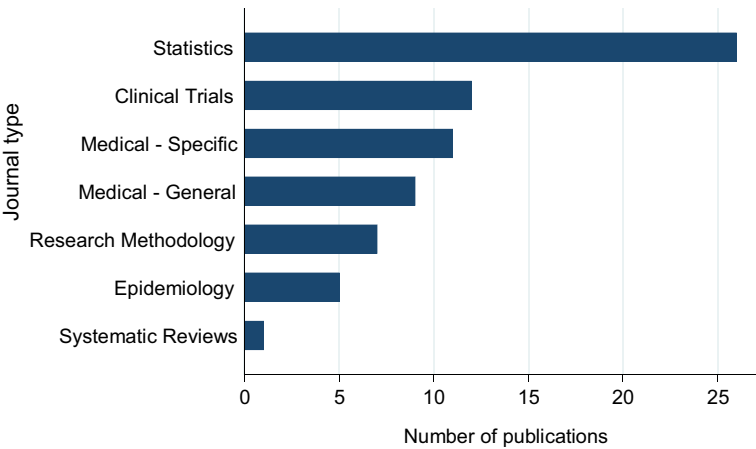

Supplement: Supplementary file 4 — Authors’ original file for figure 2 [file 13063_2014_2328_MOESM4_ESM.pdf]

Average number of citations per year

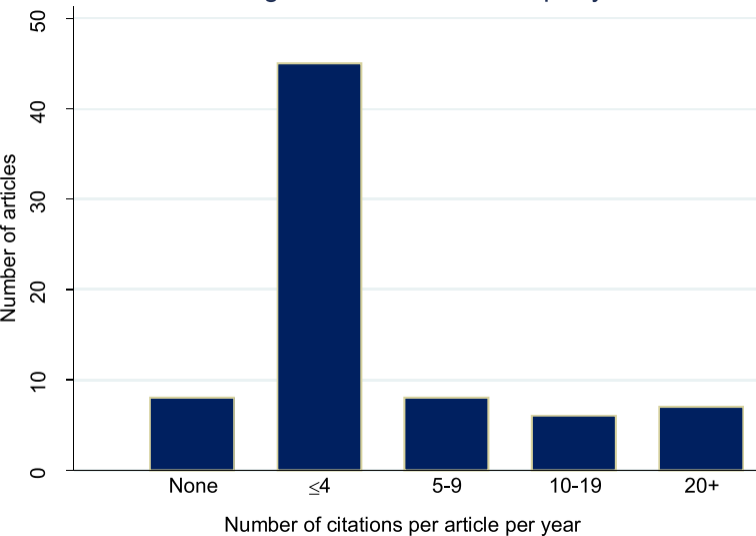

Supplement: Supplementary file 5 — Authors’ original file for figure 3 [file 13063_2014_2328_MOESM5_ESM.pdf]

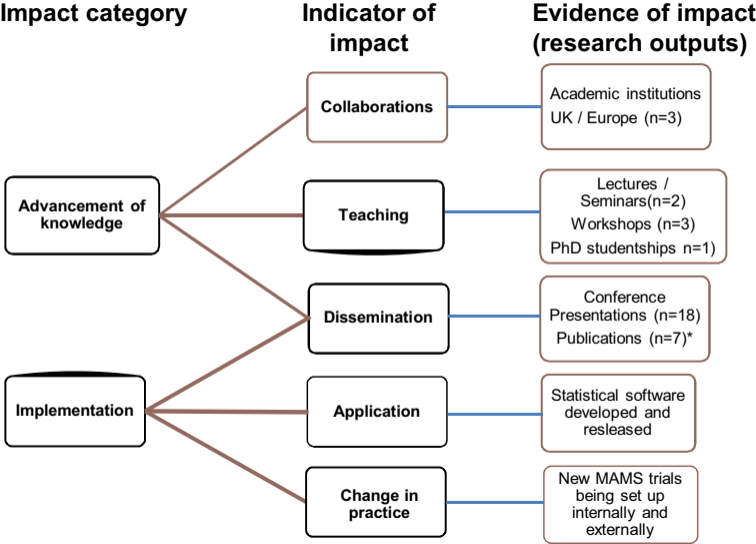

Supplement: Supplementary file 6 — Authors’ original file for figure 4 [file 13063_2014_2328_MOESM6_ESM.pdf]
